# Nanofibrous ε-Polycaprolactone Matrices Containing Nano-Hydroxyapatite and *Humulus lupulus* L. Extract: Physicochemical and Biological Characterization for Oral Applications

**DOI:** 10.3390/polym16091258

**Published:** 2024-04-30

**Authors:** Jaime Villanueva-Lumbreras, Ciro Rodriguez, María Rosa Aguilar, Hamlet Avilés-Arnaut, Geoffrey A. Cordell, Aida Rodriguez-Garcia

**Affiliations:** 1 Universidad Autónoma de Nuevo León, Facultad de Ciencias Biológicas, Instituto de Biotecnología, Ciudad Universitaria, Ave. Pedro de Alba S/N, San Nicolás de los Garza 66455, NL, Mexico; jaime.villanuevalum@gmail.com (J.V.-L.); hamlet.avilesarn@uanl.edu.mx (H.A.-A.); 2 Tecnologico de Monterrey, Escuela de Ingeniería y Ciencias, Ave. Eugenio Garza Sada 2501, Monterrey 64849, NL, Mexico; 3 Laboratorio Nacional de Manufactura Aditiva y Digital (MADIT), Apodaca 66629, NL, Mexico; 4 Instituto de Ciencia y Tecnología de Polímeros (ICTP-CSIC), 28006 Madrid, Spain; mraguilar@ictp.csic.es; 5 Networking Biomedical Research Centre in Bioengineering, Biomaterials and Nanomedicine, CIBER.BBN, 28029 Madrid, Spain; 6 Natural Products Inc., Evanston, IL 60201, USA; pharmacog@gmail.com; 7 College of Pharmacy, University of Florida, Gainesville, FL 32610, USA

**Keywords:** poly(ε-caprolactone), electrospinning, nano-hydroxyapatite, *Humulus lupulus*, hops, drug delivery system, bone regeneration

## Abstract

Oral bone defects occur as a result of trauma, cancer, infections, periodontal diseases, and caries. Autogenic and allogenic grafts are the gold standard used to treat and regenerate damaged or defective bone segments. However, these materials do not possess the antimicrobial properties necessary to inhibit the invasion of the numerous deleterious pathogens present in the oral microbiota. In the present study, poly(ε-caprolactone) (PCL), nano-hydroxyapatite (nHAp), and a commercial extract of *Humulus lupulus* L. (hops) were electrospun into polymeric matrices to assess their potential for drug delivery and bone regeneration. The fabricated matrices were analyzed using scanning electron microscopy (SEM), tensile analysis, thermogravimetric analysis (TGA), FTIR assay, and *in vitro* hydrolytic degradation. The antimicrobial properties were evaluated against the oral pathogens *Streptococcus mutans*, *Porphyromonas gingivalis*, and *Aggregatibacter actinomycetemcomitans*. The cytocompatibility was proved using the MTT assay. SEM analysis established the nanostructured matrices present in the three-dimensional interconnected network. The present research provides new information about the interaction of natural compounds with ceramic and polymeric biomaterials. The hop extract and other natural or synthetic medicinal agents can be effectively loaded into PCL fibers and have the potential to be used in oral applications.

## 1. Introduction

Advances in the field of biomaterials and nanotechnology have revolutionized traditional therapeutic modalities to improve the functionality of drug delivery and tissue engineering [1]. Biomaterials are a critical element in the development of scaffolds that provide a three-dimensional niche to mimic the extracellular matrix (ECM), and facilitate cell migration, proliferation, and vascularization, which are essential elements during tissue regeneration [2]. An adequate scaffold for bone regeneration should possess several properties relating to biocompatibility, biodegradability, porosity, and osteoinductivity [3] to promote bone growth, and to provide temporary mechanical support in the bone defect area. Biocompatibility allows the cells to adhere to a scaffold without stimulating an undesirable immune response, and to proliferate into regenerated functional tissue. That is a principal objective of tissue engineering wherein, over time, the human cells replace the implanted scaffold through the production of new tissue. A porous scaffold with adequate mechanical properties permits cell migration and the formation of a structure that can support manipulation during the surgical process and subsequently until tissue regeneration is complete [4].

Poly(ε-caprolactone) (PCL) is a biocompatible and biodegradable polymer employed as a support matrix for the fabrication of scaffolds that encompass active adjuvants [5]. It is widely used in tissue regeneration applications due to its ease of handling when applying a variety of processing techniques [6]; however, it possesses poor mechanical and osteoconductivity properties. To overcome these limitations, the incorporation of inorganic components into the polymeric matrices, such as bioactive glasses and ceramics, e.g., hydroxyapatite, improves the mechanical strength and fosters osteoconduction during the bone regeneration processes [7,8,9]. In addition, modification of the scaffolds through the incorporation of bioactive compounds improves their functionality [10], and acts as a highly targeted delivery system for medicinal agents, which stimulates and guides the cellular regeneration process while concomitantly inhibiting both microbial infection and transplant rejection [11].

Hydroxyapatite (Ca_10_(OH)_2_(PO_4_)_6_) provides important biological characteristics, including being a part of the bone matrix and promoting bone cell differentiation, which can assist during the mineralization process, and as an osteoconductive material can be used in the area requiring bone regeneration [12,13].

Bone loss in the oral cavity may occur as a result of trauma, cancer, congenital diseases, periodontal diseases, and caries [14]. Periodontitis is an infectious and inflammatory disease caused mainly by the Gram-negative bacteria *Porphyromonas gingivalis* and *Aggregatibacter actinomycetemcomitans*. It is an important disease that causes tooth loss in humans through the progressive destruction of the tissues supporting the teeth [15]. Caries, a multifactorial infectious disease, results in the demineralization of dental hard tissues and the loss of teeth. The primary causative agent associated with dental caries is *Streptococcus mutans*, a microorganism that can form biofilms [16]. A common failure of the existing scaffolds for oral applications following surgery is subsequent microbial infection, which inhibits tissue generation [17]. One possible solution is to fabricate scaffolds that, through the incorporation of antimicrobial agents, have the capacity to inhibit microbial infection [18].

Natural products incorporated into scaffolds were investigated for their synergistic action in combination with biomaterials for bone regeneration and the prevention of infection [18]. Due to the increase in microbial multidrug resistance (MDR) to many front-line antibiotics, the search for antimicrobial metabolites from plants has gained interest [19]. *Humulus lupulus* L. (known as hops) is a member of the Cannabaceae family and was originally from Western Asia. Now, it is naturalized in central Europe and is widely cultivated in North and South America, South Africa, and Australia [20]. The bitter metabolites produced by the flower heads (cones) are used in the brewing industry for their organoleptic properties that improve the flavor and aroma of beer [21]. Due to the rich composition of hop cones, they have long been used as a valuable raw material in a variety of applications. The secondary metabolites, the bitter acids, the polyphenols, and the essential oil components, have demonstrated anticancer and anti-inflammatory properties; antibacterial, antifungal, and anticancer activities; and anti-inflammatory, anticancer, and analgesic effects, respectively [20].

Extracts of hop cones were evaluated for their antimicrobial properties against oral Streptococci, particularly *S. mutans*, *S. sanguis*, and *S. salivarius* [22,23]. The principal metabolites characterized from hops: humulone, isohumulone, lupulone, and xanthohumol, have demonstrated anti-inflammatory [24], antimicrobial [25], and anticancer activities, and inhibition of bone resorption [26]. Xanthohumol, an important prenylated chalconoid derivative in the female inflorescence of hops, enhances osteoblast differentiation at concentrations of 0.001, 0.01, 0.1, and 1 µg/mL [27]. The extract used in the present research was a commercial CO_2_-prepared hop extract (Hopsteiner).

Of the fabrication techniques employed to develop scaffolds, electrospinning is a simple process that produces nanofibers considered to be a highly effective platform for biomedical applications for drug release, wound healing, and tissue engineering [28]. Electrospinning enables the combination of biomaterials to generate nanofiber membranes through the application of variable manufacturing parameters. In addition, it is a method for the fabrication of porous, three-dimensional, delivery systems of synthetic and natural compounds to introduce biological properties into the matrix for diverse applications [29,30].

This study was designed to fabricate and characterize membranes of PCL nanofibers loaded with nanohydroxyapatite and the hop extract, produced through electrospinning, and to evaluate their physicochemical, mechanical, and in vitro antimicrobial and cytotoxic properties. The originality of this study is, first, the combination of both the hop extract loaded in PCL nanofibers and the addition of the nanohydroxiapatite, aiming to develop an antibacterial system to be used against oral pathogens.

## 2. Materials and Methods

### 2.1. Materials

Poly(***ε***-caprolactone) pellets (Mw = 80,000 Da) and hydroxyapatite (nanopowder < 200 nm particle size) were purchased from Sigma Aldrich (St. Louis, MO, USA). The *Humulus lupulus* L. extract containing mainly 83.2% of α-bitter acids (humulone) and 97.3% of β-bitter acids (lupulone), and 90% of xanthohumol was obtained from the Hopsteiner Company (New York, NY, USA). The hop extract was diluted in methanol to prepare the stock solution with a final concentration of 10 mg/mL. The extract was stored at 4 °C in the dark until used. Acetone and methanol were purchased from CTR Scientific S.A de C.V (Monterrey, NL, Mexico), and syringes were purchased from Becton Dickinson (Franklin Lakes, NJ, USA).

### 2.2. Fabrication of Electrospun Nanofibers

The matrices were fabricated using an electrospinning apparatus composed of a high-voltage source of power (Gamma High Voltage Research, Ormond Beach, FL, USA), a syringe infusion pump (KD Scientific, Holliston, MA, USA) fitted with a metallic blunt-tip 27G needle (CML Supply, Lexington, KY, USA), and a 5 mL plastic syringe (BD Plastipak™, Franklin Lakes, NJ, USA). The polymeric solutions were prepared with PCL 9% *w*/*v*, using acetone as the solvent and stirred overnight at room temperature. The nanohydroxyapatite was dispersed in acetone, sonicated for 10 min, and added to the polymeric solution. The different concentrations of nanohydroxyapatite were 1, 3, and 5% *w*/*v*, while the hop extract was added in concentrations of 1, 3, and 5% *v*/*v*. The final solutions were stirred for 24 h at room temperature, then transferred into the syringe, injected with the infusion pump, and electrospun under optimized parameters (0.4 mL/h flow rate, 15 cm distance from the needle tip to the collector, and 25 kV) at room temperature and at 50% relative humidity. The fibers were collected and deposited in a 10 × 10 cm metallic collector covered by non-stick aluminum foil. During the experiments, the relative humidity was 50% and the temperature was 25 °C. The samples obtained from the electrospinning technique were dried under vacuum for 48 h to complete solvent removal. The conferred names for the samples are related to their composition. Thus, the PCL samples were composed of only PCL, the PCL + nHAp samples were composed of PCL and nanohydroxyapatite, and the PCL + nHAp + hop extract samples were composed of PCL, nanohydroxyapatite, and the hop extract.

### 2.3. Morphological Characterization

Examinations of the morphology and the overall fiber distribution of the electrospun matrices were carried out with a scanning electron microscope Philips XL30 SEM TMP (F.E.I. Company, Hillsboro, OR, USA). The samples were covered with a sputtered gold film (Polaron SC7640, Newhaven, UK) and observed in the SEM. The average fiber diameter and distribution were analyzed using ImageJ Version 1.54 software (National Institutes of Health, Bethesda, MD, USA).

### 2.4. Thermogravimetric Analysis

Thermogravimetric analysis (TGA) of the electrospun matrices was carried out in a Thermogravimetric Analyzer TA Q500 (New Castle, DE, USA). The samples were stored overnight at room temperature for 24 h, then weighted in aluminium pans and heated from room temperature to 600 °C at a heating rate of 10 °C/min^−1^ under a nitrogen atmosphere. Weight losses of the samples were measured as a function of temperature.

### 2.5. Fourier-Transform Infrared Spectroscopy (FTIR) Analysis

The functional groups present in the dried samples of PCL, nHap, and hop extracts were analyzed using FTIR analysis using a Perkin Elmer FTIR spectrometer (Waltham, MA, USA) equipped with a PIKE Miracle diamond ATR attachment at wavenumbers ranging from 4000 to 400 cm^−1^. The infrared spectra were recorded in transmission mode using nanofiber matrix deposited on a silicon wafer.

### 2.6. Mechanical Characterization

The mechanical properties (tensile strength and elongation at break) of the obtained matrices were determined with a universal testing machine (Instron 3345, Worthing, Sussex, UK). Rectangular samples (3 mm × 10 mm) were cut and mounted between holders and tested (*n* = 8) under dry conditions and at 20 °C. Mechanical data were obtained from the stress–strain curves of each sample and expressed in MPa. The results were reported as mean ± standard deviation (SD).

### 2.7. In Vitro Degradation Assays

The degradation tests were performed in accordance with ISO 10993-13: 2010 [31]. Triplicate samples (~10 mg) of the electrospun matrices were immersed in sterile flasks with 100 mL of phosphate-buffered saline (PBS) at pH ~7.4 at 37 °C, with constant agitation at 120 rpm for 8 weeks. After each immersion period was concluded, the samples were removed and washed three times with distilled water to remove salt residues, dried under vacuum to constant mass and weighed.

The percentage weight loss was calculated using the following equation:

$$Weight\;loss\;(\%)=\frac{\left(Wi-Wf\right)}{Wi}\times 100$$

where *Wi* is the initial sample weight and *Wf* is the final sample weight after degradation at different time periods.

### 2.8. Antibacterial Assay

The *in vitro* antibacterial activity of the acquired hop extract was evaluated against the oral pathogenic organisms *Streptococcus mutans* (Sm) (ATCC 700610), *Porphyromonas gingivalis* (Pg) (ATCC 33277), and *Aggregatibacter actinomycetemcomitans* (Aa) (ATCC 43718) through measuring the zones of inhibition using the agar diffusion assay [32]. *S. mutans* was cultured on brain heart infusion (BHI) (Becton Dickinson, Sparks, MD, USA) for 24 h at 37 °C and 5% CO_2_, while *P. gingivalis* and *A. actinomycetemcomitans* were cultured on tryptic soy broth (TSB) (Becton Dickinson, Monterrey, NL, Mexico) for 24 h at 37 °C under anaerobic conditions. Microbial suspensions with an optical density of 0.5 McFarland standard at 1.5 × 10^8^ CFU/mL (CFU: colony forming unit) were prepared in sterile 0.9% NaCl.

Before testing, the minimum inhibitory concentration (MIC) of the hop extract was determined using a turbidity assay by inoculating a bacterial suspension into BHI broth (10 mL) to provide an initial density of 1 × 10^5^ CFU/mL. Concentrations of the hop extract ranging from 0.1 µg/mL to 150 µg/mL were tested. The tubes with each strain were incubated at 37 °C for 24 h and growth inhibition was determined spectrophotometrically at 410 nm using a Multiscan Ascent Microplate Reader (Thermo Fisher Scientific, Waltham, MA, USA). All experiments were performed in triplicate.

#### 2.8.1. Determination of Growth Inhibition Zones of the *H. lupulus* Extract

The hop extract was tested initially for its antibacterial activity using the disc diffusion method. Disc samples (6 mm diameter) were prepared from sterile filter paper and impregnated with the hop extract (100 µL) corresponding to 50 µg/disc, then dried for 24 h at room temperature. The discs were placed on the agar surface inoculated with the individual bacterial strain and the plates incubated at 37 °C for 24 h depending on the strain used in the test, as indicated previously. After the incubation period, the diameters of the growth inhibition zones were measured using calipers and recorded. Chlorhexidine gluconate 0.12% (Sigma-Aldrich, St. Louis, MO, USA) was used as the positive control and saline solution as the negative control. Tests were repeated three times to assess reproducibility.

#### 2.8.2. Determination of Growth Inhibition Zones of the Matrices

For the antibacterial activity of the matrices, disc samples (6 mm in diameter) were cut from the electrospun matrices and sterilized by UV radiation for 30 min before placing on the cultured agar plates. The discs of the PCL/nHAp/hop extract were placed on Petri dishes containing the requisite agar, inoculated with the corresponding microorganisms, *S. mutans*, *P. gingivalis*, or *A. actinomycetemcomitans*, and incubated at 35 °C for 24 h under anaerobic conditions. The inhibition zones (mm) were measured for each sample. Chlorhexidine gluconate 0.12% was used as the positive control and saline solution as the negative control. All the tests were conducted independently in triplicate experiments.

### 2.9. Cell Culture and Cell Viability Assay

The viability test was carried out with the Detroit 548 CCL-116 cell line of human skin fibroblasts using the direct contact assay method and following the International Organization for Standardization (1993) protocols [33]. Cells (1 × 10^4^) were seeded in 96-well microplates and incubated for 24 h at 37 °C in a 5% CO_2_ atmosphere in DMEM (Dulbecco’s Modified Eagle Medium) (Gibco, Grand Island, NY, USA) supplemented with 10% fetal bovine serum (Gibco). All the processed matrices were cut into round discs of 5 mm and sterilized under UV light for 30 min, soaked in 70% ethanol for 30 min, then washed twice with PBS.

The sterile disc samples were placed in the 96 wells along with the fibroblast cells and incubated in DMEM for 48 h at 37 °C in a 5% CO_2_ atmosphere. After this period, nanofiber samples were removed from the wells and MTT (3-(4,5-dimethylthiazol-2-yl)-2,5-diphenyltetrazolium bromide) reagent (10 µL) was added. After incubation of the cells with MTT for 4 h, the medium with the reagent was withdrawn, and DMSO (100 µL) was added to dissolve the formazan. The optical density (OD) was measured at 570 nm with a Biotek Synergy 2 plate spectrophotometer (Winooski, VT, USA). Cells in contact with the matrices were used as a positive control, and medium with untreated cells was the blank.

The cell viability was calculated according to the following equation:

Cell viability (%) = [(OD of treated cells (with the matrices) − OD of blank)/(OD of untreated cells − OD of blank)] × 100.

All tests were performed five times in three independent experiments. 

### 2.10. Statistical Analysis

Results are shown as the mean value ± the standard deviation (SD) of the mean. ANOVA analyses of variance followed by a Tukey test were used to compare the differences between groups. Probability values of *p* ≤ 0.05 were considered significant. The statistical analysis was performed using IBM SPSS 25.0 (Corporation Business Analytics Software, Chicago, IL, USA) software.

## 3. Results

### 3.1. Morphological Characterization

**Figure 1:**
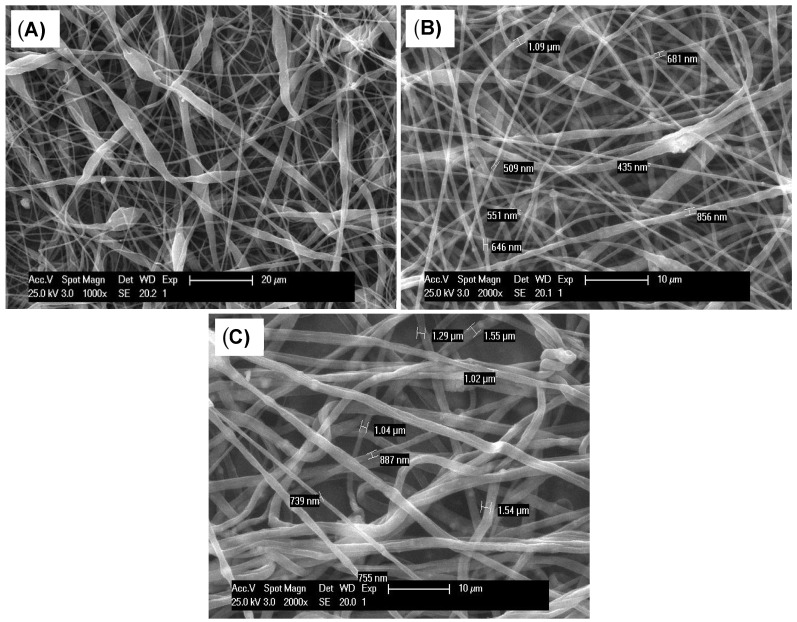
SEM micrographs showing the diameter and distribution of the electrospun fibers of PCL, nHAp, and hop extract: (**A**) PCL; (**B**) PCL + nHAp; and (**C**) PCL + nHAp + hop extract. Median fibers were calculated using more than 100 random fiber positions in three different SEM images.

The optimum sample parameters obtained in this work were a polymer concentration of 9%, an nHAp concentration of 3%, and a *Humulus lupulus* concentration of 3%. The optimized electrospinning parameters were 25 kV voltage, 15 cm distance, and 0.4 mL/h^−1^ flow rate. SEM was used to examine the morphological characteristics of the electrospun nanofibers. According to the statistical analysis, the diameter of the fibers varied based on the presence of the nHAp and the amount of hop extract introduced. All the prepared PCL, PCL + nHAp, and PCL + nHAp + hop extract samples presented a homogeneous diameter distribution of the fibers (Figure 1A–C). According to the SEM micrographs, non-aligned fiber distribution with an interconnected porosity was observed for all the samples, whereas the presence of nHAp resulted in the occurrence of some bead-like structures (Figure 1B,C).

From a medicinal perspective, the electrospinning technique has promoted the development of matrices or scaffolds with plant extracts encapsulated into polymeric nanofibers [34,35]. The processing conditions, such as the polymeric solution viscosity and conductivity, flow rate, voltage, the distance between the nozzle and the collector, and the relative humidity each affect the formation of the nanofibers. Moreover, even though the viscosity of the polymer solution or the polymer blend was not determined, the increased viscosity due to the hop extract and the nHAp might have contributed to the occurrence of the fiber diameter variation. 

Small water droplets present in the atmosphere can also interact with the polymer jet during the process. As a result, these parameters can influence the diameter, morphology, porosity, structure, and mechanical properties of the electrospun fibers [36,37]. In this study, the median size of the PCL fibers without nHAp or the hop extract was 549 nm, while the median diameter of the samples of the PCL and nHAp fibers was 681 nm, and for the fibers with the PCL/nHAp/hop extract the median diameter was 1102 nm. A study by Chong et al. [38] showed that the average diameter of PCL fibers without nHAp was 478.5 nm, and with 3% nHAp was increased to 521.1 nm. The statistical analysis performed on the selected SEM samples shows the average diameter of the fibers and the standard deviation. Tukey’s test confirmed that there were significant differences among the samples analyzed (Table 1) and demonstrated that the incorporation of the hop extract and the nHAp significantly increased the median diameter of the fibers.

Interestingly, the addition of nHAp into the PCL-based solution substantially modified the morphology of the fibers, which became wider compared to the other matrices, probably due to the agglomeration of nHAp particles on the surface and inner structure of the fibers (Figure 1B). In an earlier study by Hassan and Sultana, the PCL membranes showed a fiber diameter of 0.25 ± 0.08 µm, but when 10% of nHAp was added, the fiber diameter increased to an average of 0.65 ± 0.18 µm [39]. This study is in agreement, wherein an increased fiber diameter was observed when nHAp was added.

### 3.2. Thermogravimetric Analysis (TGA)

**Figure 2:**
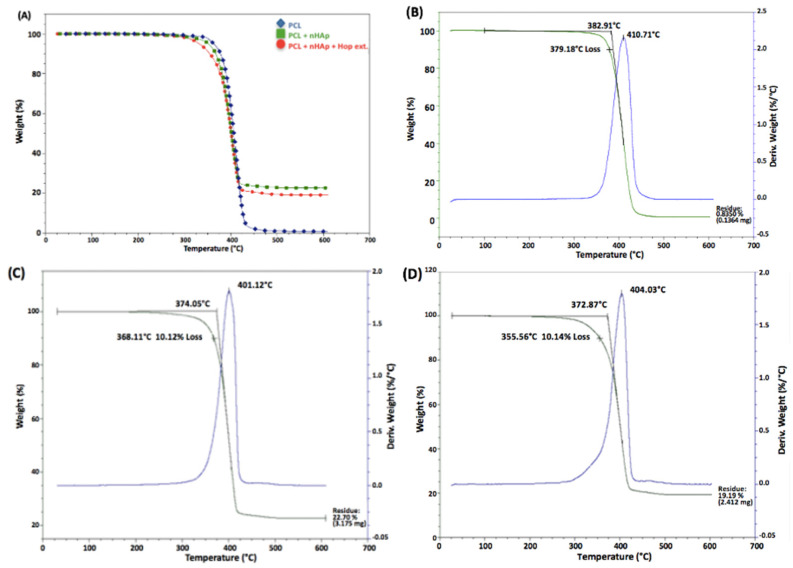
Thermographs of the analyzed matrices. (**A**) The blue line is for the PCL matrices, the green line is for the PCL + nHAp matrices, and the red line is for the PCL + nHAp + hop extract matrices; (**B**) degradation curves of PCL; (**C**) degradation curves of PCL + nHAp; and (**D**) degradation curves of PCL + nHAp + hop extract.

The thermogravimetric analyses (TGAs) are shown in Figure 2 and summarized in Table 2. The residual mass of PCL showed a one-step degradation curve and a maximum degradation temperature of 411 °C. This result was consistent with previous TGA studies of PCL degradation which presented depolymerization as a single step process since it breaches the thermal stability of PCL [40,41]. The thermal stability shown by nHAp in this study is consistent with previous studies where HAp has degradation temperatures ranging from 1000 to 1500 °C [42,43]. Variations in the maximum degradation temperature were attributed to the nHAp concentration. The low nHAp concentration used in this work (3% *w*/*v*) increased the PCL thermal degradation, as previously suggested [44,45]. PCL nanofibers manufactured with nHAp presented the highest (22.7%) post-analysis residue compared with the PCL alone and PCL/nHAp/hop samples (0.835% and 19.19%, respectively). In this study, PCL presented a residue of less than 1%, as observed previously [46]. The degradation curves of the analyzed samples are presented in Figure 2.

The thermogravimetric analysis of the samples with the hop extract and nHAp shows changes in their onset temperature, maximum weight loss temperatures, and the percentage of waste obtained from the fibers at the end of the analysis (Table 2).

### 3.3. FTIR Analysis

**Figure 3:**
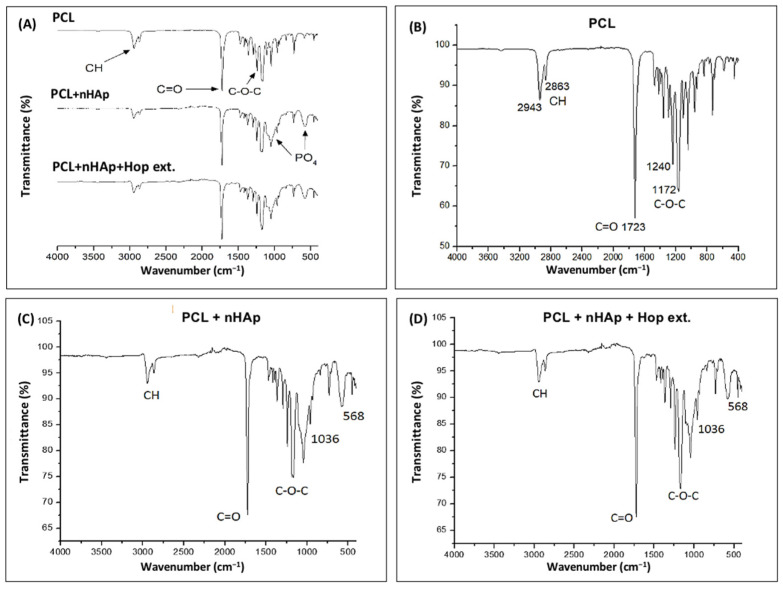
FTIR spectra for the electrospun matrices. (**A**): comparison between all the samples; (**B**): FTIR spectrum of PCL; (**C**): FTIR spectrum of PCL + nHAp; and (**D**): FTIR spectrum of PCL + nHAp + hop extract. The arrows show the characteristic absorptions of each material and the functional group assignments.

The Fourier-transform infrared (FTIR) spectra of the different combinations of electrospun matrices are shown in Figure 3A. The parent spectrum shows absorption bands at 2943 cm^−1^ and 3000 cm^−1^ that correspond to the C-H stretching vibrations of aromatic and aliphatic compounds. The absorption bands at 1240 cm^−1^ and 1723 cm^−1^ correspond to a stretching vibration for the carbonyl (C=O), carbon-hydrogen and (-CH), and C-O-C bonds, respectively, characteristic of PCL [47] (Figure 3B). Furthermore, the major absorptions of a phosphate group (PO4) identified at 1021 cm^−1^ and 564 cm^−1^ were characteristic of nHAp [48]. The infrared spectrum of PCL with nHAp showed similar characteristic peaks (Figure 3B,C).

In the FTIR spectrum of the nanofiber samples with the hop extract, a carbonyl band (C=O) occurred at 1667 cm^−1^ and at 1600 cm^−1^ for the cyclohexadienone nucleus corresponding to the major compounds of hop: humulone and lupulone [49]. The relatively low intensity of these bands (Figure 3D) is attributed predominantly to the low concentration of hop extract in the matrices, and partially to the molecular interactions between the polymer and the hop extract.

### 3.4. In Vitro Degradation Analysis

**Figure 4:**
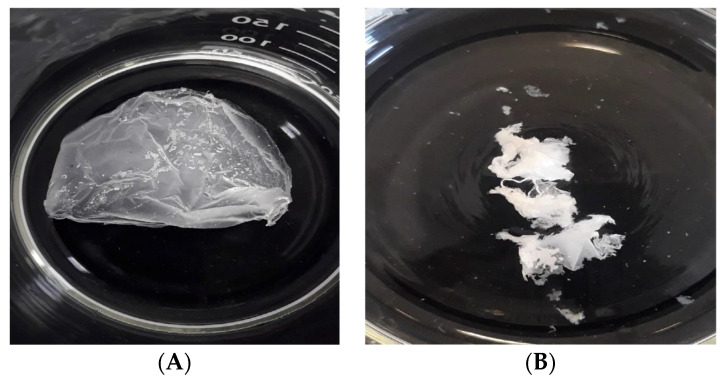
Electrospun matrices. (**A**) PCL-only sample, (**B**) PCL + nHAp + hop extract sample after 8 weeks of immersion in PBS at 37 °C with constant agitation at 120 rpm and a pH of 7.4.

The hydrolytic degradation test was conducted through immersion of the samples into sterile flasks containing 100 mL of PBS at 37 °C with constant agitation at 120 rpm (to simulate physiological conditions) for 8 weeks. The fibers that only contained PCL showed a 1% mass loss percentage after 8 weeks. Figure 4 shows the aspects of the two samples following the immersion period.

The in vitro degradation rate was consistent with a previous study of PCL characterization where molecular weight (Mw 80,000), crystallinity, pH, and temperature were influential factors [50]. The fibers with PCL, nHAp, and the hop extract showed degradation rates of 1%, 2%, and 4%, respectively, after 8 weeks, as shown in Table 3. The incorporation of both nHAp and the hop extract causes water diffusion into the amorphous regions of PCL increasing its hydrolytic degradation. Consequently, more extensive hydrolysis of nanofibers occurred compared to those produced with PCL alone [51], although ANOVA analysis showed that there were no significant differences between the degradation rates of the analyzed samples. The weight difference was observed from the start of the trial until after 8 weeks. Following that period, the samples were washed with distilled water and dried for 24 h at 40 °C and then weighed to constant weight.

### 3.5. Mechanical Characterization

The electrospun matrices were submitted to the tensile strength assay. The results of the Young’s modulus and the percentage of elongation to failure are summarized in Table 4. For the sample matrices of PCL alone, PCL + nHAp, and PCL + nHAp + hop extract, the highest values for the mechanical properties were observed for the PCL matrices alone; lower mechanical characteristics were determined for the samples of PCL + nHAp. These variations in the mechanical properties depend on the electrospinning parameters, the diameter and orientation of the fibers, the molecular weight, and the polymer concentration [52]. The addition of nHAp increases the diameter of the PCL nanofibers and decreases the tensile strength. This occurs when the amount of PCL matrices binds the nHAp particles and the stress is transferred between them [53].

In a previous study [54], the tensile strength values for dry PCL scaffolds with oriented nanofibers were 3.8 ± 0.8 MPa and an elongation at a break of 170%, while the present results showed a tensile strength and elongation at a break of 1.74 ± 0.63 and 3.8%, respectively. The differences between the previous study and this work could be due to the orientation of the fibers (aligned vs. non-aligned), the porosity of the fibers, the diameter of the fibers (250–700 nm—549 nm), and the interactions between the fibers [54].

The results obtained with PCL and nHAp are consistent with a previous study [55] where, by increasing the amount of hydroxyapatite, the percentage tensile deformation of the nanofibers decreased. Other research [56] also demonstrated that compared with fibers without any plant extract, fibers including a plant extract had a decrease in Young’s modulus and variation in the percentage of tensile deformation. This phenomenon also depends on the properties of the natural compounds incorporated into the fibers [57,58], and the increase or decrease in these parameters may be attributed to the different chemical interactions of the metabolites with the polymer [56]. The ANOVA analysis shows that there is no significant difference in the tensile deformation between the analyzed samples.

### 3.6. Antibacterial Assay

In the present studies, the commercial *H. lupulus* extract was evaluated at 3% against Gram-positive (*Streptococcocus mutans*) and Gram-negative periodontal pathogens (*P. gingivalis* and *A. actinomycetemcomitans*). The antimicrobial activity of the commercial Hopsteiner *Humulus lupulus* extract against oral Gram-positive microorganisms was demonstrated in previous studies [59,60], while it was inactive against oral Gram-negative pathogens [61], with the exception of *Phorphyromonas gingivalis* [25]. The MIC for *S. mutans* was assessed at ≤32 μg/mL while *P. gingivalis* presented a MIC at ≤64 μg/mL, whereas *A. actinomycetemcomitans* was resistant to *H. lupulus* (MICs ≥ 150 μg/mL). Previous data have shown that *H. lupulus* possesses antimicrobial activity against *Streptococcocus* spp. with a minimum inhibitory concentration (MIC) of 0.39 µg/mL [62].

#### 3.6.1. Determination of Growth Inhibition Zones

The disc diffusion method is a sensitive and standardized test to quantify the ability of antibiotics to inhibit bacterial growth. In this work, a commercial extract of *H. lupulus* that contains α-acids, β-acids, and other compounds was used. 

It was suggested that the flavonoids and polyphenols contained in the *H. lupulus* extracts, such as the α- and β-acids and xanthohumol, are likely responsible for the growth inhibition of Gram-positive [63] and Gram-negative bacteria [64]. A possible mechanism of the antibacterial activity of hops might include the leakage of the cell membrane due to the hydrophobic nature of the β acids and their high pKa that facilitate their entrance through the bacterial membrane, decreasing the intracellular pH, reducing the lactic acid production, and causing cell wall lysis [65].

The present study provides evidence for the antibacterial effects of the hop extract on the oral pathogenic organisms *S. mutans*, *P. gingivalis*, and *A. actinomycetemcomitans* using filter paper discs impregnated with the hop extract (100 µL). The results showed inhibition zone diameters of 19 mm for *S. mutans*, 15 mm for *P. gingivalis*, and 12 mm for *A. actinomycetemcomitans*. Also, in this study, all the strains showed growth inhibition when they were exposed to 0.12% chlorhexidine gluconate with halo zones of 28 mm, 25 mm, and 23 mm, respectively. The zones of bacterial growth inhibition are summarized in Table 5.

In the studies of Gregory et al. [63], ten different commercial hop extracts were evaluated against *Streptococcus mutans* biofilm formation. The best results were for two extracts that showed a significant inhibition of biofilm formation at a 1:256 dilution [63]. Another study described the protective activity of a hop extract on the viability of periodontal ligament cells when they were infected with *P. gingivalis* [66]. In addition, a clinical study performed on human volunteers showed that the use of a mouth rinse containing a 0.1% hop polyphenol mixture applied five times a day significantly decreased the accumulation of microbial plaque on the surface of the teeth [67].

#### 3.6.2. Determination of Growth Inhibition Zones of the Matrices

As indicated in Table 5, the results from the matrices showed zones of growth inhibition of 4 mm for *S. mutans*, 2 mm for *P. gingivalis*, and no activity against *A. actinomycetemcomitans*. This very low antimicrobial activity against the oral pathogens could be attributed to the low amount (3% *v*/*v*) of hop extract used to fabricate the polymeric matrices. In another study, higher concentrations of the hop extract were used to obtain an antimicrobial effect [59]. Another possible cause for the weak antimicrobial activity of the matrices against the oral pathogens may be that the hop metabolites can be degraded and oxidized with UV irradiation during the sterilization process [68] or the release of the extract from the matrix may be limited.

### 3.7. Cytotoxicity Assay

The most common solvents used to dissolve polycaprolactone are dichloromethane (DCM), trifluoroethanol, and dimethyl formamide (DMF). However, these solvents are toxic to the upper respiratory tract and eyes and require safety precautions when used during the electrospinning process [68]. That was the most important reason to utilize acetone in this work to avoid those risks. The cytotoxicity assessments of the samples were performed using human fibroblasts and applying the MTT assay.

**Figure 5:**
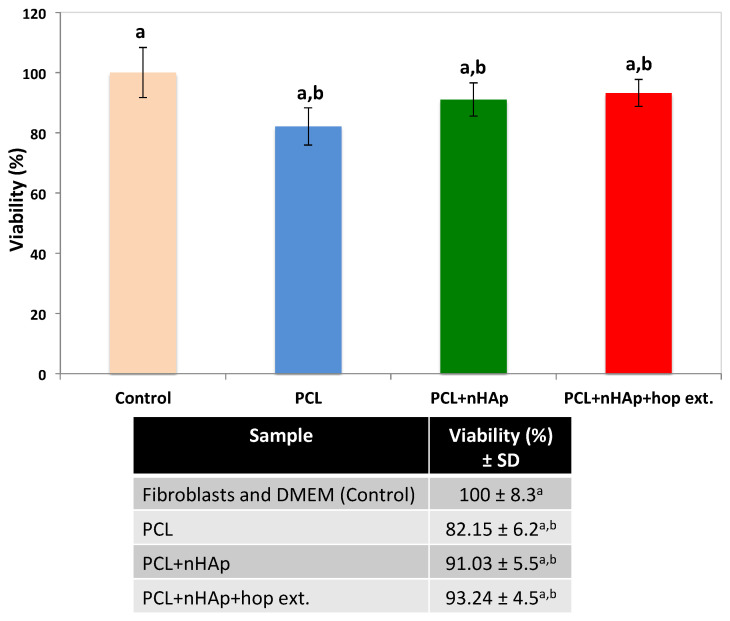
Graph and inset table showing the mean and standard deviation (±SD) of the percentage viability (*N* = 4) of the human skin fibroblasts cultured on the electrospun matrices. Distinct lowercase letters after the standard deviation indicate statistically significant differences between the matrice samples (*p* < 0.05).

All of the samples tested in this work showed non-cytotoxic activity, with equal viability percentages of the human skin fibroblasts after being in contact with the matrices. This confirms previous research where the PCL and nHAp nanofibers were shown to be non-cytotoxic, biocompatible materials [69,70]. Additionally, the fibers containing the *H. lupulus* extract did not show any cytotoxic effects on human skin fibroblasts after 48 h exposure, indicating that they represent favorable cell biocompatible materials, and confirming the previously reported results [71]. Figure 5 shows the viability of the fibroblasts after being exposed to the PCL + nHAp + hop extract matrices. According to ISO 10993-5, each of the matrices presented a cell viability higher than 70%, which means that all were cytocompatible [33].

The limitations of the study include a need to establish the stability of the metabolites of *H. lupulus* through metabolomics and analyzing the release of the metabolites from the matrix in the biological assays. Another limitation in achieving controlled drug delivery under in vitro conditions is that PCL presents a low degradation and slow drug release due to its high hydrophobicity. To overcome those limitations, we propose the PCL surface modification and encapsulation of the hop extract into liposomes or nanoparticles to prevent the loss of the compound release and to improve their bioavailability. Also, the evaluation of the degree of PCL + nHAp crystallinity must be performed to evaluate their mechanical properties and degradation behavior.

## 4. Conclusions

This study demonstrated that nHAp and the *H. lupulus* extract can be successfully co-incorporated into PCL electrospun fibers. The mixture of PCL, nHAp, and the *H. lupulus* extract showed differences in physicochemical properties, indicating that the properties of the matrix can be modulated by adding different concentrations of nHAp and/or the hop extract. However, the low concentration of hop extract (3%) incorporated into the PCL matrices only showed weak antimicrobial activity against *S. mutans* and *P. gingivalis* and did not inhibit the growth of *A. actinomycetemcomitans*. Increasing the concentration of the hop extract and evaluating the kinetic release of its constituents from the PCL matrices through HPLC chromatography over a two-month period to determine if the extract release is sustained are proposed for further studies.

The incorporation of the extract and nHAp and the elaboration of the electrospun nanofibers open a window of possibilities for the fabrication of membranes with natural compounds as potential materials for the delivery of medicinal agents and for oral bone tissue regeneration in various applications once the delivery of an effective dose of the bioactive material can be realized. 

## Figures and Tables

**Table 1:** Median fiber diameter (in nm) of the electrospun matrices.

Sample	Fiber Diameter (nm ± SD)	*p* Value
PCL	549 ± 60 ^c^	0.70
PCL + nHAp	681 ± 112 ^b^	0.99
PCL + nHAp + hop ext.	1102 ± 162 ^a^	0.01

Distinct superscript letters indicate statistically significant differences among groups (*p* < 0.05).

**Table 2:** Onset temperatures, maximum weight loss temperature, and residue of the processed electrospun matrices.

Sample	Onset (°C)	Max. Weight Loss Temp (°C)	Residue (%)
PCL	382.91	410.71	0.83
PCL + nHAp	374.05	401.12	22.70
PCL + nHAp + hop ext.	372.87	404.03	19.20

**Table 3:** Differences in weight loss of the electrospun matrices following the 8-week degradation period.

Sample	Initial Weight (mg)	Final Weight (mg)	Weight Loss (%)
PCL	10.13	10.02	1
PCL + nHAp	10.10	9.90	2
PCL + nHAp + hop ext.	10.13	9.70	4

**Table 4:** Young’s modulus values for the electrospun samples and the percentage of elongation.

Sample	Tensile Strength (MPa) (Mean ± SD)	Elongation at Break (% ± SD)
PCL	1.74 ± 0.63 ^a^	3.8 ± 0.66 ^a^
PCL + nHAp	1.39 ± 0.69 ^a^	2.2 ± 0.81 ^b^
PCL + nHAp + hop ext.	1.07 ± 0.35 ^a^	5.4 ± 1.90 ^a^

Distinct superscript letters indicate statistically significant differences among groups (*p*< 0.05).

**Table 5:** Zones of bacterial growth inhibition (mm) produced by chlorhexidine, the hop extract, and the PCL matrices with nHAp and with hop extract.

Sample			Inhibition Zone (mm) ± SD
	*Sm*	*Pg*	*Aa*
Chlorhexidine 0.12% (+control)	28 ± 0.4 ^a^	25 ± 2.1 ^a^	23 ± 0.4 ^a^
Hop extract	19 ± 0.5 ^b^	15 ± 0.8 ^b^	12 ± 4.4 ^b^
PCL + nHAp + hop ext.	4 ± 1.2 ^c^	2 ± 1.7 ^c^	0 ± 0.5 ^c^

Distinct superscript letters indicate statistically significant differences among groups (*p* < 0.05).

## Data Availability

Data are contained within the article.

## Abbreviations

FTIR	Fourier-transform infrared spectroscopy
Hop	*Humulus lupulus* L.
nHAp	Nano-hydroxyapatite
PBS	Phosphate-buffered saline
PCL	Poly(ε-caprolactone)
SEM	Scanning Electron Microscope
TGA	Thermogravimetric Analysis (TGA)
